# 

**Published:** 1874-05

**Authors:** 


### Books and Pamphlets Received.

A Practicle Treatise on the Surgical Diseases of the Genito-Urinary Organs, including Syphilis. Designed as a Manual for Students and Practitioners. With Engravings and Cases. By W. C. Van Buren, A. M., M. D., and E. L. Keyes, A. M., M. D. New York: D. Appleton & Co., 1874. Buffalo: Herger & Ulrich.

Lectures on the Diseases of Infancy and Childhood. By Charles West, M. D. Fifth American, from the Sixth Enlarged, and Revised English Edition. Philadelphia. Henry C Lea, 1874. Buffalo: Theo. Butler & Son.

The Science of Homœopathy; or, A Critical and Synthetical Exposition of the Doctrines of the Homœopathic School. By Charles J. Hemple, M. D. New York: Boericke & Tafel.

Syphilitic Membranoid Occlusion of the Rima Glotidis. By Louis Elsberg, M. D. From American Journal of Syphelography January, 1874.

Coal as a Reservoir of Power. By Robt. Hunt, F. R. S.. Atoms. By Prof. M. D. No. 11 of Half-Hour Recreations in Popular Science. Boston: Estes & Lauriat. Buffalo: Martin Taylor.

School Hygiene; A paper read by R. J. O'Sullivan, M. D., before the N. Y. Academy of Medicine, June 19th, 1873.

Observations on the Pathology and Treatment of Cholera. The result of Forty Years Experience. By John Murray, M. D. New York: G. P. Putnam's Sons, 1874. Buffalo: Martin Taylor.

Urethrotomy, External and Internal Combined, in cases of Multiple and Difficult Stricture; with remarks on the Urethral Calibre. By F. N. Otis, Mr. D.. From New York Med. Journal, April 1874. New York: D. Appleton & Co.

On Intra-Uterine Fibroids. By J. Marion Sims, M. D. From New York Medical Journal April 1874. New York: D. Appleton & Co.

The Mutual Relations of Druggists and Physicians. An Address before the Graduating Class of the Massachusetts College of Pharmacy. By Charles E. Buckingham, M D. From Boston Medical and Surgical Journal. Boston: David Clapp & Son.

The Conditions of the Conflict. An Oration delivered before the Medical Society of the County of Kings, at its Fifty-Second Annivesary, February 24th, 1874. By Alexander Hutchins, M. D.

### Medical and Surgical Journal

PUBLISHED MONTHLY,

Containing Original Articles, Reports of Medical Societies and Hospitals, Editorial, Reviews, Correspondence, Army News, etc., etc ; including the usual variety of Medical Periodical Publications. Specimen copies sent on application.

Address Buffalo Medical & Surgical Journal, Buffalo, N. Y.

TERMS OF SUBSCRIPTION, - - $3.00 PER YEAR, IN ADVANCE.

